# Metabolic Syndrome: Updates on Pathophysiology and Management in 2021

**DOI:** 10.3390/ijms23020786

**Published:** 2022-01-12

**Authors:** Gracia Fahed, Laurence Aoun, Morgan Bou Zerdan, Sabine Allam, Maroun Bou Zerdan, Youssef Bouferraa, Hazem I. Assi

**Affiliations:** 1 Faculty of Medicine, American University of Beirut, Beirut 2020, Lebanon; gif04@mail.aub.edu (G.F.); morganzerdan@gmail.com (M.B.Z.); 2 Faculty of Medicine and Medical Sciences, Holy Spirit University of Kaslik (USEK), Jounieh 1200, Lebanon; Laurenceaoun90@gmail.com; 3 Faculty of Medicine, University of Balamand, Beirut 1100, Lebanon; sabineallam@hotmail.com; 4 Department of Internal Medicine, SUNY Upstate Medical University, New York, NY 13205, USA; marounzerdan@gmail.com; 5 Department of Internal Medicine, Cleveland Clinic Foundation, Cleveland, OH 44195, USA; youssefbf95@gmail.com; 6 Department of Internal Medicine, Division of Hematology and Oncology, Naef K. Basile Cancer Institute, American University of Beirut Medical Center, Beirut 2020, Lebanon

**Keywords:** metabolic syndrome, insulin resistance, nutraceuticals

## Abstract

Metabolic syndrome (MetS) forms a cluster of metabolic dysregulations including insulin resistance, atherogenic dyslipidemia, central obesity, and hypertension. The pathogenesis of MetS encompasses multiple genetic and acquired entities that fall under the umbrella of insulin resistance and chronic low-grade inflammation. If left untreated, MetS is significantly associated with an increased risk of developing diabetes and cardiovascular diseases (CVDs). Given that CVDs constitute by far the leading cause of morbidity and mortality worldwide, it has become essential to investigate the role played by MetS in this context to reduce the heavy burden of the disease. As such, and while MetS relatively constitutes a novel clinical entity, the extent of research about the disease has been exponentially growing in the past few decades. However, many aspects of this clinical entity are still not completely understood, and many questions remain unanswered to date. In this review, we provide a historical background and highlight the epidemiology of MetS. We also discuss the current and latest knowledge about the histopathology and pathophysiology of the disease. Finally, we summarize the most recent updates about the management and the prevention of this clinical syndrome.

## 1. Introduction

### 1.1. History

In 1988, Reaven raised the possibility that insulin resistance (IR) is not only involved in the etiology of type 2 diabetes mellitus (T2DM) but also that of cardiovascular disease (CVD) [1]. Reaven remarked that IR frequently presents in conjunction with a set of abnormalities and described them as syndrome X. The qualifier “metabolic” was added to Reaven’s syndrome X to differentiate it from the pre-existing syndrome X in cardiology [2]. In sum, metabolic syndrome X is a risk factor for cardiovascular diseases (CVDs) even without concomitant T2DM and it includes IR, hyperinsulinemia, dysglycemia, dyslipidemia, and hypertension. The disorders are, respectively, assessed using six indices to make the diagnosis of metabolic syndrome (MetS): waist circumference, fasting glucose levels, triglyceride levels, high-density lipoprotein (HDL) levels, cholesterol levels, and blood pressure (Table 1) [2,3].

Notice that blood insulin is not part of the five criteria because measuring this parameter for large-scale screening is cumbersome in clinical practice. Waist circumference is employed instead, as it was later proven to strongly correlate with IR [9]. Total body fat did not initially take part in the characterization of Reaven’s MetS as he found many cases showing a reverse association between total body fat and IR. Subgroup analyses later revealed that body fat distribution was the missing element in the equation explaining heterogeneity in results [10]. “Apple-shaped” bodies demonstrate central or abdominal visceral obesity and therefore carry a greater risk of developing IR than individuals with “pear-shaped” bodies with subcutaneous fat accumulation [11,12]. However, waist circumference alone remains inconclusive of abdominal adiposity and must be interpreted along with body mass index (BMI) in order to promptly evaluate for the high-risk abdominal obesity [9]. In addition, excess visceral adipose tissue is often accompanied by fat infiltration of hepatocytes, a condition known as non-alcoholic fatty liver. The latter is a dangerous phenotype of the disease, possibly progressing to cirrhosis or liver cancer [13]. 

Not a disease per se, but a term that serves as an umbrella of risk factors for individuals to be at an increased risk of disease, MetS has appeared for the first time on PubMed in 1940. Alberti et al. described a global definition of MetS in 2009 where three abnormal findings would qualify a person for MetS. Risk factors such as raised blood pressure, dyslipidemia (raised triglycerides and lowered high-density lipoprotein cholesterol), raised fasting glucose, and central obesity were proposed by the International Diabetes Federation and the American Heart Association/National Heart, Lung, and Blood Institute [14]. 

A single set of cut points was used for all components except waist circumference, for which national or regional cut points were utilized. Several studies later on have shown that waist-to-height ratio was a better screening tool than waist circumference [14,15]. 

### 1.2. Epidemiology

According to the Center of Disease Control and Prevention (CDC), the United States witnessed a 35% increase in MetS prevalence since the appearance of the term in the 1980s till 2012 [16]. The incidence of MetS matches that of obesity and T2DM. About 85% of T2DM patients also have MetS and thus are at higher risk of CVDs [14]. As such, in 2017, about 12.2% of the USA adult population had T2DM. Around one-quarter of them were unaware of their disease. Unsurprisingly, the MetS prevalence was three times higher, marking about one-third of the American adult population [4]. Fortunately, the National Health and Nutrition Examination Survey (NHANES) released recent data demonstrating declining numbers of the disease with 24% in men and 22% in women [17].

## 2. Pathophysiology

### 2.1. Established Ideas 

**Figure 1:**
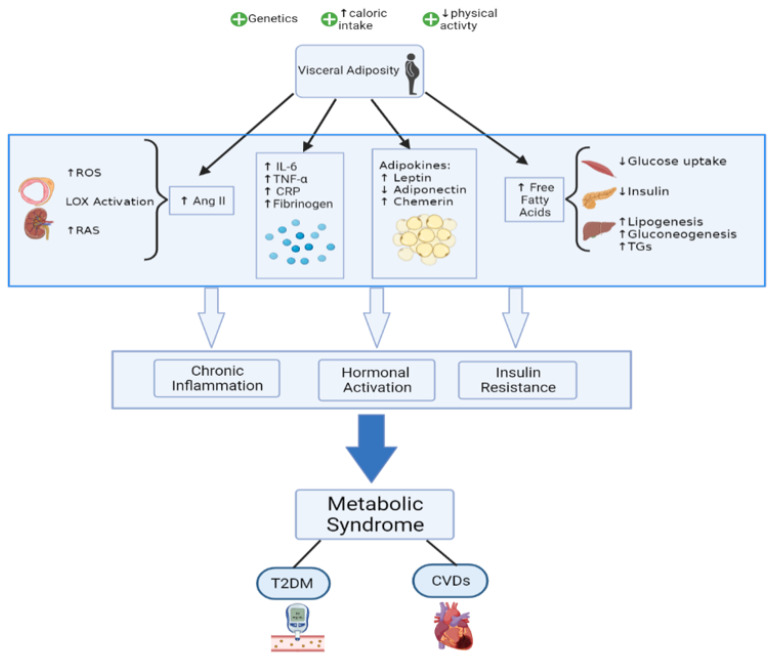
Mechanisms highlighting MetS pathophysiology.

The pathophysiology of the MetS encompasses several complex mechanisms that are yet to be fully elucidated. It is still debated as to whether the different elements of MetS form by themselves distinct pathologies or fall under a common, broader pathogenic process. In addition to genetic and epigenetic factors [18], some lifestyle and environmental such as overeating and lack of physical activity have been identified as major contributors to the development of MetS. A causative role can be given to high caloric intake since visceral adiposity has been shown to be an important trigger that activates most of the pathways of MetS [19,20]. Among the proposed mechanisms, insulin resistance, chronic inflammation, and neurohormonal activation seem to be essential players in the progression of MetS and its subsequent transition to CVDs and T2DM (Figure 1).

#### 2.1.1. Insulin Resistance

Insulin, a peptide hormone secreted by the pancreatic beta cells in response to high blood glucose, exerts its anabolic effects by inhibiting lipolysis and hepatic gluconeogenesis, while increasing glucose uptake in liver, muscles, and adipose tissues. When insulin resistance develops in fat tissues, insulin-mediated inhibition of lipolysis is impaired. The resulting increase in circulating free fatty acids (FFAs) in turn worsens insulin resistance by causing alterations in the insulin signaling cascade in different organs, thus creating a vicious cycle [21,22]. In muscles, FFAs affect insulin receptor substrate (IRS-1)-associated PI3K activity, leading to decreased GLUT-4 translocation to the surface and hence reduced glucose uptake [22]. In parallel, FFAs act on the liver to promote gluconeogenesis and lipogenesis. The net result is a hyperinsulinemic state to maintain normal glucose levels. However, the compensation eventually fails, leading to a decrease in insulin levels, which is further exacerbated by the lipotoxic effect of FFAs on beta cells of the pancreas [21,23]. It is essential to note that visceral lipolysis increases the supply of FFAs directly to the liver via the splanchnic circulation, thus making visceral fat deposits more important contributors to insulin resistance than subcutaneous fat [24]. 

In addition, high concentrations of FFAs increase cholesterol esters and triglyceride (TG) synthesis and subsequently the production of very low-density lipoproteins (VLDLs) rich in TGs. These in turn activate cholesterol ester transfer protein (CETP), which promotes the TGs transfer from VLDL to HDL, increasing HDL clearance and decreasing its concentrations [25]. Moreover, triglyceride-rich LDL, formed after exchange for LDL cholesterol ester, becomes hydrolyzed by lipoprotein or hepatic lipase, leading to cholesterol-depleted small dense LDL particles (Sd-LDL) [26]. All these alterations in lipoproteins concentrations constitute the hallmark of the atherogenic dyslipidemia caused by insulin resistance in MetS.

Another contribution of insulin resistance to MetS is the development of hypertension caused partly by loss of insulin’s vasodilatory effect and by FFA-induced vasoconstriction due to reactive oxygen species production and subsequent scavenging of nitric oxide [27]. Other mechanisms involve increased sympathetic stimulation and renin-induced sodium reabsorption in the kidneys [28]. Moreover, insulin resistance leads to higher serum viscosity, creates a prothrombotic state, and increases pro-inflammatory cytokine release from the adipose tissue, all of which play an important role in increasing the risks of CVDs and T2DM [29].

The best way to diagnose insulin resistance was known to be euglycemic hyperinsulinemic; however, many simple, cost effective and minimally invasive technics were established to assess a good predictive accuracy of this condition [30,31,32,33]. Utilizing these technics, simply using the fasting blood glucose and insulin levels, homeostatic model assessment–insulin resistance (HOMA-IR) index and quantitative insulin sensitivity check index (QUICKI) can be calculated. According to Hrebicek et al. (2002) and Singh and Saxena (2010), these two indices have been shown to be valuable and very helpful tools in evaluating insulin resistance in epidemiological studies and clinical practices.

This table demonstrates the area under the curve (AUC) of insulin resistance indices and their optimal cutoff points for diagnosis of MetS by sex. According to the results of Motamed et al. [34], the optimal cutoff points for HOMA-IR in the diagnosis of MetS in men was 2.0 (YI = 0.312) and in women was 2.5 (YI = 0.255). The optimal cutoff points for diagnosis of MetS for QUICKI was 0.343 (YI = 0.315) in men and 0.331 (YI = 0.264) in women. The AUC curves of insulin resistance indices and their optimal cutoff points for diagnosis of MetS can be found in Table 2.

#### 2.1.2. Adipose Tissue, an Endocrine Organ

Aside from being a thermoregulator and lipid storage facility, the recently discovered endocrine function of the adipose tissue provides additional mechanistic understandings to the development of MetS [35]. The various adipokines released include hormones (e.g., leptin, adiponectin), peptides (e.g., angiotensinogen, apelin, resistin, and plasminogen activator inhibitor (PAI)-1), and inflammatory cytokines (e.g., interleukin (IL)-6, tumor necrosis factor α (TNFα), visfatin, omentin, and chemerin), all of which play a major role in the pathophysiology of insulin resistance and MetS [36] (Table 2). Among the hormones released, leptin levels were shown to be directly proportional to obesity and body fat levels [37]. When body energy stores are adequate, leptin suppresses food intake and stimulates energy expenditure while also controlling glucose homeostasis and insulin sensitivity [38]. However, the failure of high leptin levels to correct the metabolic imbalance seen in obesity has given rise to the concept of “leptin resistance”, in which tissues have decreased sensitivity to leptin [39]. Moreover, leptin is known to promote a proinflammatory immune response as it was shown to activate the Th1 pathway and reverse the starvation-induced immunosuppression [40]. Since higher leptin levels correlate with increased cardiovascular risk and inflammation, leptin is suggested to be an important factor linking obesity, MetS, and CVDs [41]. 

In contrast, adiponectin’s effects counter those of leptin as it was established as an anti-atherogenic, anti-inflammatory, and anti-diabetic adipokine [42,43]. These properties emanate from the fact that adiponectin affects the nuclear factor kappa-light-chain enhancer of activated B cells (NF-kB) inflammatory pathway [44], increases insulin sensitivity [45], inhibits vascular smooth muscle cell (VSMC) proliferation, and stabilizes plaque formation [46]. Several studies have shown decreased adiponectin levels in patients with coronary heart disease [47,48], diabetes [49], and hypertension [50] compared to control subjects, thus conferring a protective role for adiponectin against the development and progression of insulin resistance, high blood pressure, and CVDs. In fact, individuals with reported genetic hypoadiponectinemia caused by a missense mutation demonstrate higher tendency to develop MetS [51].

In addition to the dysregulation of leptin and adiponectin levels in MetS, chemerin, a well characterized chemoattractant abundantly produced by adipocytes, has recently gained attention as a potential MetS biomarker after its suggested involvement in inflammation, glucose metabolism, adipogenesis, and angiogenesis in animal models and cell culture studies [52,53]. In humans, several small cohort studies revealed a significant increase in circulating chemerin levels in individuals fulfilling MetS criteria [54,55,56], and this significance persisted even after adjusting for waist circumference or BMI, since obesity itself is known to increase chemerin levels [57]. Although Chu et al. [58] report that high chemerin with low adiponectin increase the risk of MetS with an odds ratio of 5.8, a recent report by Shafer-Eggleton et al. [59] suggests that while the chemerin/HDL-C ratio can be a superior biomarker and better predictor of nascent MetS than hsCRP, the chemerin/adiponectin ratio is not significant after adjustment for waist circumference [59]. Larger studies need to confirm these findings and establish the true relationship between chemerin levels and MetS. 

Another important neurohormonal pathway in the development of MetS is the involvement of the renin–angiotensinogen system (RAS). As mentioned earlier, the adipose tissue produces the peptide angiotensin II (Ang II) after activation of angiotensin-converting enzyme. Plasma Ang II levels were shown to be increased in obesity and insulin resistance [60]. The peptide exerts its pathogenic effects through activation of nicotinamide adenine dinucleotide phosphate (NADPH) oxidase, which increases the production of reactive oxygen species (ROS) [61,62]. ROS has multiple pleiotropic effects, including endothelial injury, expression of NF-kB, platelet aggregation, oxidation of LDL, and expression of lipoprotein receptor-1 (LOX-1) on VSMCs and endothelium. Together, RAS, LOX-1, and ROS form a positive feedback loop and induce a vicious cycle of endothelial dysfunction, inflammation, and fibroblast proliferation, leading to the progression of dyslipidemia, T2DM, hypertension, vasculopathies, and CVDs [63,64,65].

#### 2.1.3. Chronic Inflammation

The various pathogenic pathways contributing to the development of MetS culminate in a pro-inflammatory state that explains the elevation in various inflammatory markers such as IL-6, C-reactive protein (CRP), and TNFα seen in individuals with MetS [66] (Table 3). As mentioned earlier, insulin resistance and obesity-induced systemic oxidant stress activates downstream inflammatory cascades, leading to tissue fibrosis, atherogenesis, and subsequently CVDs [67]. 

IL-6 is one of the cytokines released by both macrophages and adipocytes [68], and its levels were shown to be increased with insulin resistance and obesity. In fact, IL-6 is known to regulate fat and glucose metabolism, mediating insulin resistance by way of different complex mechanisms [69]. 

This cytokine acts on various tissues, leading to the metabolic effects of obesity. In the liver, IL-6 increases the production of acute phase reactants including CRP. High levels of CRP were demonstrated in several studies to have the strongest correlation with cardiac events, T2DM, and MetS [70]. IL-6 also favors a prothrombotic state by increasing fibrinogen levels, another acute phase reactant [71]. Moreover, IL-6 targets other tissues such as VSMCs and endothelial cells to promote the expression of vascular cell adhesion molecules (VCAMs) and local RAS pathway activation, leading to vascular wall atherosclerosis, inflammation, and dysfunction [72]. 

TNFα is another cytokine produced within the adipose tissue, mainly from the local macrophages, and its production also varies proportionally with adipose tissue mass and correlates with insulin resistance, both being major features of MetS [68,73]. TNFα exerts its pathogenic effects by impairing insulin signaling in adipocytes and hepatocytes through serine phosphorylation and inactivation of insulin receptors and downstream signaling molecules, resulting in diminished metabolic effects of insulin [74]. TNFα also contributes to insulin resistance by inducing hepatic lipolysis, thus increasing FFA levels in the circulation [75]. 

Other players contributing to the chronic inflammatory state seen in MetS are the innate immune system receptors such as Toll-like receptors (TLRs). TLRs are involved in pathogen recognition and modulate the innate immune response by activating downstream inflammatory signaling pathways that lead to the release of various cytokines (TNFα, IL-6, IL-1β, and monocyte chemoattractant protein-1 (MCP-1)) [76]. The immune response is initiated after TLR recognition of ligands such as pathogen-associated molecular patterns (PAMPs) emanating from pathogens (e.g., LPS) and damage-associated molecular patterns (DAMPs) derived from damaged inflamed host tissues. Among the DAMPs, many endogenous ligands such as saturated fatty acids (SFAs), modified LDLs, advanced glycation end-products, extracellular matrix degradation products, and heat shock proteins, are recognized by TLRs, especially TLR2 and TLR4, activating a pro-inflammatory cascade [76,77]. 

Several animal and human studies have highlighted the role of TLRs in MetS. In mouse models with diet induced MetS, TLR4 expression and activity was shown to be increased. In addition, protection from high-fat diet-induced insulin resistance was conferred to TLR4 KO mice with lesser tissue inflammation observed [78]. Similarly, when compared to wild-type mice, TLR2 deficient models exhibited diminished insulin resistance, adiposity, macrophage infiltration, and cytokine expression in adipose tissue [79]. 

In humans, a rise in mRNA levels and a significant increase in expression of both TLR2 and TLR4 on the surface of monocytes has been demonstrated in subjects with MetS, even after accounting for waist circumference [80]. Similar findings were observed by Hardy et al. [81] in adolescents with MetS when compared to BMI-matched controls. This increase in innate immune receptor activation has been correlated with an increasing level of both endogenous (fatty acids, Ox-LDL, lipopolysaccharide-binding protein, etc.) and exogenous (LPS) activators in patients with MetS [80,82]. The latter finding of higher endotoxin (LPS) levels in MetS is intriguing as it supports the hypothesis of gut microbiota perturbation in MetS, resulting in increased gut permeability [83] and higher levels of LPS, the classical ligand of TLR4 [80]. 

Thus, a thorough understanding of TLRs involvement in MetS would help establish a new selective therapeutic target which can alleviate the burden of the chronic inflammation present in MetS. 

### 2.2. Novel Studies

The complex interplay of environmental factors, lifestyle, and genetic/epigenetic factors in the pathophysiology of MetS has led to the emergence of novel studies that evaluate new perspectives in the early diagnosis, classification of new biomarkers, and discovery of potential targets for therapeutic interventions. 

#### 2.2.1. Fetuin-A

Fetuin-A, also referred to as α2-Heremans–Schmid glycoprotein (AHSG), is a protein with pleiotropic metabolic effects secreted by the liver. In addition to being a hepatokine, fetuin-A has recently been described as a potential adipokine since its expression and secretion levels have been also shown to be increased in visceral adipose tissue of obese animal models [84] and humans with MetS [85]. The association of its circulating levels with MetS is currently gaining importance in the literature as a novel biomarker and possible risk factor for the development of MetS [86]. A recently published meta-analysis of 14 eligible studies showed that circulating fetuin-A levels are significantly higher in patients with MetS compared to controls, with a possible trend towards an increase in the risk of MetS with an increase in the circulating fetuin-A concentrations [87]. One mechanistic approach is the contribution of fetuin-A to insulin resistance, as demonstrated in several animal [88,89] and human [90] studies. In fact, fetuin-A inhibits the tyrosine kinase activity of the insulin receptor as well as IRS-1 phosphorylation and downstream molecules in the PI3k/akt pathway [88]. These effects were reversed in fetuin-A knockout mice, which exhibited enhanced insulin sensitivity, glucose clearance, and less serum FFAs and triglycerides [91]. Furthermore, fetuin-A mediates macrophage migration and infiltration into the adipose tissue by way of chemo-attractants, thus inducing inflammatory cytokine release and subsequently contributing to the development of MetS as discussed earlier [92]. 

In terms of regulation, the elevated concentrations of FFAs seen in patients with MetS induce the binding of the inflammatory protein NF-κB to fetuin-A promoter, hence increasing circulating fetuin-A mRNA expression, protein synthesis, and secretion [93]. In contrast, adiponectin has recently been shown to have an inhibitory effect on the expression of fetuin-A, which can partly explain the increased circulating fetuin-A concentrations in hypoadiponectinemia seen with the MetS [94]. 

Aside from being supported by experimental and clinical studies, the link between circulating fetuin-A and MetS has also been proposed by genetic studies. The human fetuin-A or the AHSG gene is located on chromosome 3q27, which has been identified as T2DM susceptibility locus [95] and has been mapped as a quantitative trait locus for MetS [96]. Moreover, single-nucleotide polymorphisms (SNPs) of the AHSG gene and their relationship to MetS features such as BMI have been reported in several studies [97]. Taken together, it appears that the association between circulating fetuin-A and MetS is facilitated at least partially by genetics. 

#### 2.2.2. Mitochondrial Dysfunction and PGC-1α in MetS

Aside from its essential role in ATP production, mitochondria are a major source of ROS generation due to the leak of few high energy electrons from the electron transport chain (ETC) and their direct reaction with oxygen [98]. However, mitochondria are equipped with a very effective antioxidant mechanism that activates superoxide dismutase and other enzymes to scavenge ROS produced either locally or by other organelles like the peroxisomes [99,100]. Thus, mitochondrial dysfunction can lead to ROS overproduction causing cell injury, the hallmark “oxidative stress” that contributes to the development of many disease processes including MetS [101]. 

In turn, several components of the MetS can be responsible for the excessive ROS production that overwhelms mitochondrial antioxidant capacity and leads to its dysfunction. On one hand, the adipocyte nutrient excess seen in individuals with MetS result in a compensatory upregulation of fatty acid oxidation that increases NADH and FADH2 load from the tricarboxylic acid cycle, providing more electron supply to the ETC. Spilling of some of these high-energy electrons off the chain produces excessive ROS and subsequently exhausts and damages the mitochondrial antioxidant defense mechanisms [102,103]. Moreover, the excess FFAs in adipocytes activate NADPH oxidase, which increases ROS generation [104]. 

On the other hand, insulin resistance may also contribute to the decline in mitochondrial function. Insulin-resistant cells exhibit increased susceptibility to oxidative stress and reduced oxidative phosphorylation and energy production [105]. 

In addition, the decreased energy requirements due to the sedentary lifestyle seen in MetS reduces the levels of peroxisome proliferator-activated receptor gamma coactivator −1 α (PGC-1α) and thus decreases mitochondrial biogenesis. PGC-1α is described as an essential transcriptional activator and master regulator of mitochondrial biogenesis and function, particularly oxidative phosphorylation, and ROS detoxification [106]. Mitochondrial biogenesis pathways are activated in response to high-energy requirements translated by increased AMP:ADP/ATP and NAD+:NADH ratios, which are mediated by AMP-activated protein kinase (AMPK) and Sirtuin-1 pathways, respectively [107]. Increased expression of PGC-1α in energy-demanding tissues leads to the activation of the transcription factors estrogen related receptors (ERRs) and nuclear respiratory factors 1 and 2 (NRF-1 and NRF-2), which in turn induce the mitochondrial transcription factor A (TFAM) [108]. TFAM, along with the mitochondrial transcription factor B2 (TFB2M), activate the transcription of mitochondrial genes, leading to increased mitochondrial mass [109]. In addition, PGC-1α enhances fatty acid oxidation as it co-activates peroxisome proliferator-activated receptor α and δ (PPARα and PPARδ), responsible for the expression of mitochondrial fatty acid oxidation genes [110]. PGC-1α also increases the expression of antioxidant enzymes in the mitochondria, such as catalase, manganese superoxide dismutase, and heme oxygenase, leading to less oxidative stress and less mitochondrial dysfunction [111,112]. 

Therefore, any dysregulation in PGC-1α activity may alter the metabolic function of tissues, leading to the development of various metabolic diseases. In fact, SNPs in the human PGC-1α gene was demonstrated to be associated with diabetes, obesity, and hypertension, major components of the MetS [113]. Research interest in PGC-1α and its correlation with MetS is substantially growing since PGC-1α can be a potential therapeutic target against MetS [114,115] as it connects mitochondrial metabolism with redox homeostasis that is found to be dysfunctional in metabolic diseases. 

As mentioned above, NADPH oxidase plays an essential role in the generation of ROS. It has been shown that the activation of the NADPH oxidase system increases the production of ROS, contributing to the pathogenesis of atherosclerosis, diabetes, and cardiovascular diseases [116]. Jialal et al. examined the difference in the expression of two subunits of NADPH oxidase (p22 phox and p47 phox) between participants with and without MetS and concluded that both subunits were significantly increased in participants with MetS as compared to controls [117]. While previous studies have shown an increase in the p22 phox subunit only, this is the first study to show an increase in both subunits [117,118]. This is likely because this study recruited patients with nascent MetS, and participants with diabetes mellitus or on statin therapy were excluded [117,118]. 

#### 2.2.3. Circulatory MicroRNAs

MicroRNAs (miRNAs) are small evolutionarily conserved noncoding RNAs with various biological functions, mainly negatively regulating post-transcriptional gene expression [119]. In mammals, miRNAs control around 60% of all protein-coding genes [120]. Circulatory microRNAs (c-miRNAs) have gained enormous research interest because of the underscored significance of their expression profiles and regulatory functions in many diseases, as well as their putative use as biomarkers for diagnosis, progression, and disease prognosis [121,122]. The role of c-miRNAs in metabolism regulation and cardio-metabolic disorder development has been recently studied in both animal and human models. In fact, a dysregulation in the c-miRNA expression profile is demonstrated to be linked to different components of the MetS [123,124]. For example, miR-17-5p and miR-519d are related to obesity and lipogenesis [125,126], miR-375 controls insulin secretion from the pancreas [127], whereas the let-7 family affect insulin sensitivity and regulate various players in glucose metabolism [128]. Moreover, a differential miRNA expression between sexes has been reported, and this might help explain sex differences attributed to the progression to MetS and its complications [129,130]. 

A recent study by Ramzan et al. analyzed participants’ plasma expression of 26 miRNAs selected on the basis of evidence from multiple studies as being essential in the regulation of main components of cardio-metabolic diseases. The authors characterized different expression of panels and examined their relationship with early stage MetS before the progression to T2DM or CVDs [131]. Among these 26 miRNAs, 16 miRNAs were sex-specific, and 10 miRNAs were found to have altered circulating levels in participants diagnosed as having MetS, compared to healthy controls. Two of them, miR-17-5p and miR-15a-5p, were found to be the strongest predictors of MetS presence compared to healthy controls, as their expression panel was decreased in individuals with MetS, independent of sex [131]. Following putative target gene analysis [132], the two c-miRNAs had a robust regulatory communication with cellular pathways (fatty acid metabolism, AMPK, wnt, insulin, and TGF-β signaling) involved in the development of cardiometabolic diseases [133]. In addition to regulating insulin secretion and pancreatic β-cell proliferation and adaptation to metabolic stress [134], miR-17-5p was reported by Ramzan et al. to have a negative association with BMI and waist circumference, which was in concordance to previous studies [125], thus confirming the potential correlation between miR-17-5p and central adiposity, an important feature of MetS. Similarly, the pancreatic-specific miR-15a-5p was previously identified to interact with various signaling pathways such as insulin secretion and glucose metabolism [135], pancreatic inflammation [136], angiogenesis, and endothelial dysfunction [137]. The downregulation of miR-15a-5p in MetS was suggested in the study by Ramzan et al. to be mostly associated with increased total circulating TGs and visceral fat in both men and women [131]. As for the differential expression of miRNAs based on sex, this study featured a uniform downregulation in the let-7 miRNA family (let-7a-5p, miR-7c-5p, miR-7d-5p, and miR-7e-5p) in MetS men when compared to healthy controls [131]. This difference was not evident in women with MetS, which makes the let-7 family a sex-specific biomarker for MetS in men and hence can be further studied to delineate the sex differences attributed to the development and progression of MetS. 

It is important to note that the miRNA expression panel modification is not only associated with the disease itself, but also shown to be induced by dietary interventions [138]. In a recent randomized clinical trial, Marsetti et al. analyzed the expression panel of miRNAs of white blood cells (WBCs) after administering each group of MetS participants two hypocaloric diets (traditional Mediterranean diet (MD) and the American Heart Association (AHA) dietary recommendations) for 8 weeks [139]. MD was previously suggested to reduce proinflammatory markers together with inducing weight loss [140], and similarly the AHA dietary recommendations were proven to promote health benefits, lower body fat, and ameliorate some features of the MetS [141]. In their trial, Marsetti et al. reported a significant change in the expression of a total of 49 miRNAs after 8 weeks of the weight loss dietary interventions. In particular, the most relevant changes were the downregulation of miR-214 and miR-190 expression panel and the upregulation of miR-410 and miR-637 expression panel. Moreover, miR-2115, miR-587, and miR-96 showed differential expressions after 8 weeks between the MD and AHA recommended diet [139]. Interestingly, the study highlights an association between miRNA expression modifications with biochemical and anthropometric parameters such as BMI and leptin (particularly with miR-410) and VCAM-1 (particularly with miR-587). This relationship suggests that these miRNAs might be directly or indirectly involved in inducing the effects of hypocaloric diets with different macronutrient composition, which subsequently regulate metabolic disease progression. Hence, in addition to being easy-to-measure disease biomarkers [142], miRNA expression panels can be potentially used as nutritional biomarkers to monitor response to dietary interventions. 

#### 2.2.4. IDEFICS/I.Family Study and the Underlying Genetic Predisposition for MetS Risk

Since the prevalence of MetS in children and young adult is on the rise [143,144], understanding the genetics behind MetS and their effect on the MetS network are essential for early detection of people with high genetic risk of disease development throughout their life. This provides a critical insight into the etiology of the disease and might encourage individuals at high risk to adopt lifestyle changes to reduce this risk. Nagrani et al. recently studied the link between common genetic variants and repeated MetS score of children and adolescents enrolled in the IDEFICS/I.Family cohort study with meta-analysis [145]. The IDEFICS (Identification and Prevention of Dietary- and Lifestyle-Induced Health Effects in Children and Infants) is a large multicenter population-based European cohort study that aims to explore the causes and prevent the development of diet and lifestyle-related diseases in children and adolescents with a special focus on overweight and obesity [146]. Baseline measures and survey of 16,224 children aged 2–9 years were collected with a repeated follow up after two years. A third wave of follow-up and measurements is done after six years by the I.Family study, creating a longitudinal database for the young population and their families [147]. 

Various studies reported common genetic loci associated with MetS at a single time-point using case–control or cross-sectional designs [148,149]. However, Nagrani et al. were the first to explore the longitudinal association of 350 pre-selected genetic loci with a MetS score calculated continuously throughout the transition from childhood to adolescence in the IDEFICS/I.Family cohort of children. Their results were confirmed with meta-analysis of identified SNPs [145]. A significant association affecting the repeated MetS score is seen in five SNPs of the fat mass and obesity associated (FTO) gene on chromosomal region 16q12., the strongest of which was with the variant rs80540136. This is supported by other studies, whereby variations of the FTO genes have been reported as contributors to childhood and adult obesity [150], as well as T2DM [151], both being features of the MetS. The nuclear protein coded by the FTO gene belongs to the superfamily of non-heme iron and 2-oxoglutarate-dependent oxygenases and is mainly expressed in the hypothalamus where it plays a fundamental role in regulation of food intake and energy homeostasis [152]. Its expression in the liver has recently gained attention and found to modify gene expression implicated in lipid and glucose metabolism [153]. 

In conclusion, we can infer that gene of obesity and lipid metabolism drive the genetic predisposition to MetS development, and some variants contribute to a more severe MetS phenotype (higher MetS score) than others. Accordingly, devising polygenic risk scores for MetS that include the associated genetic variants might be valuable for identifying high-risk groups in children and subsequently adults, thus allowing for prevention and earlier targeted interventions. 

#### 2.2.5. GPCRs Gene Expression Affected by MetS Etiology

As discussed previously, MetS development consists of an interplay between genetic, epigenetic, and environmental factors. Interestingly, gene expression of certain proteins has been recently shown to vary according to the MetS etiology. For example, Romero-Nava et al. highlight the differential genetic expression of two types of G protein-coupled receptors (GPCRs) caused by environmental and genetic etiologies of MetS by comparing gene expression in Wistar rats fed with 70% fructose for 9 weeks, serving as a diet-induced MetS model, and Zucker obese rats serving as the genetic model [154]. The studied receptors belong to the orphan receptors, a new class of GPCRs whose endogenous ligand was initially unknown. Orphan receptors’ expression had been demonstrated to change in different pathologies, such as GPR22 and GPR162 in diabetes [155], and GPR3, GPR6, and GPR12 in dyslipidemia [156].

As for their association with MetS, Romero-Nava et al. evaluated the gene expression of GPR21 and GPR82 in the heart, liver, kidney, brain, and aorta in both the diet rat models and genetic models in comparison with the controls. In fact, GPR21 has been suggested to play a role in affecting body weight, insulin sensitivity, and glucose tolerance as GPR21 KO mice have improved insulin response and are protected from obesity [157]. In the two MetS models (diet and genetic-induced) of Romero-Nava et al., GPR21 had different pattern of expression in different tissues. In the liver, a decrease in expression was reported in fructose-treated rats, contrarily to the increase seen in Zucker obese rats. This difference might have been due to the participation of this receptor in glucose metabolism in the liver and the fact that fructose-fed rats are overloaded with carbohydrates. No difference in expression was noted in the brain; however, the two models share the same pattern of decreased expression in the kidney, aorta, and the heart compared to controls. This hints that the GPR21 receptor could be involved in hypertension seen in MetS independently of its etiology since these three tissues are important for blood pressure control [154]. GPR21 mediates its signaling mechanism by Gq protein activation [158], which is known to form the second messengers diacylglycerol (DAG) and inositol 1,4,5-triphosphate (IP3) responsible for increasing intracellular calcium and vasoconstriction at the vascular level, promoting cardiac hypertrophy at the cardiac level and controlling glomerular filtration rate in the kidneys [159,160]. We postulate that the decrease GPR21 expression in the aorta, heart, and kidneys of the diet and genetic MetS models is a compensation towards lowering the high blood pressure observed in MetS. 

GPR82 is another orphan receptor involved in metabolism, as GPR82-deficient mice were shown to decrease body weight, fat content, and TGs levels and to increase glucose tolerance and insulin sensitivity, all of which are associated with influences on food intake rather than modified respiratory and metabolic rates [161]. Although GPR82 receptors also mediate their effect through coupling to Gq proteins, their expression and effects vary according to tissue type and depend on whether the MetS model is diet or genetically induced [154]. For example, in the fructose-treated rats, their expression was decreased in the brain, kidney, and liver; increased in the heart; and not changed in the aorta. However, obese Zucker rats had an increased expression of GPR82 in the liver and brain but a decreased expression in the heart, aorta, and kidney.

The described results highlight that the differential expression of GPR21 and GPR82 receptors in various tissues is not only associated with the MetS but also depends on the etiology of the syndrome, thus being selective candidates for new therapeutic targets of metabolic diseases depending on their cause. 

#### 2.2.6. Parental MetS Epigenetic Effects on Offspring

Although the pathophysiology and the consequences of MetS on the patients have been extensively studied in the literature, little is known about the impact of maternal and/or paternal MetS on disease risk modification in offspring. Interestingly, it has been postulated that parental epigenetic alterations could be transferred to the next generation and thus reprogram the hepatic lipid metabolism of the offspring, contributing in particular to the development of non-alcoholic fatty liver disease (NAFLD) [162,163]. 

Animal models are being used to test this hypothesis linking parental MetS to NAFLD priming. For example, De Jesus et al. used a liver-specific insulin receptor knockout (LIKRO) mouse model that manifests three hallmarks of MetS: dyslipidemia, hyperglycemia, and insulin resistance [164,165]. Their results demonstrate that maternal and paternal MetS affect hepatic methylation status of members of the TGF-β superfamily, particularly the neuronal regeneration-related protein (NREP) and growth differentiation factor 15 (GDF15), which in turn alter the expression of many genes regulating hepatic lipid metabolism [166]. In fact, the offspring of either maternal or paternal LIKRO mice showed remarkable exacerbation in hepatic steatosis after administration of a high-fat diet [166]. 

One mechanistic explanation to NAFLD priming by De Jesus et al. suggests that downregulation of NREP increases protein expression of ATP-citrate lyase (ACLY) and 3-hydroxy-3-methylglutaryl-CoA reductase (HMGCR) [166], two enzymes involved in cholesterol and fatty acids biosynthesis [167]. This ACLY and HMGCR activation was demonstrated to be Akt (protein kinase B)-dependent in response to TGF-β signaling. In addition to previous studies that report that NREP exerts its action through inhibiting TGF-β1 and TGF-β2 receptor levels and decreasing TGF-β autoinduction by binding to the latency-associated protein (LAP) of TGF-β1 and TGF-β2 [168], we can infer that NREP decreases HMGCR and ACLY levels by altering Akt signaling via the noncanonical TGF-β pathway. 

De Jesus et al. extrapolated their results to human subjects by reporting decreased hepatic NREP expression in patients with NAFLD, a significant negative correlation between NREP and ACLY hepatic mRNA levels, and an important association between low-serum NREP concentrations with steatosis grade and NAFL activity score [166]. 

Altogether, the results of this novel study highlight the contribution of parental MetS to the epigenome of the next generation by widespread DNA methylation modification and shed light on potential therapeutic targets to prevent disease progression and its subsequent complications in the clinic. 

#### 2.2.7. Metabolomics

The role of various metabolites in the pathogenesis of MetS has also been recently assessed [169]. In fact, biogenic amines that are usually found in red meats, including choline, L-carnitine, and trimethylamine-N-oxide, were shown to be associated with an adverse cardiometabolic profile and insulin resistance [169,170,171,172]. In addition, several amino acids, including alanine, glutamate, glutamine, aspartate and asparagine, arginine, histidine, methionine, cysteine, and lysine have been shown to play a role in the pathogenesis of MetS [169]. For example, branch chain amino acids and alanine have been shown to be involved in insulin resistance development [173]. In addition, together with aromatic amines (phenylalanine, tryptophan, tyrosine, and phospholipids), branch chain amino acids can even serve as biomarkers to predict the start of MetS in patients who have not yet developed diabetes [174,175,176]. On the other side, histidine and lysine were shown to have antioxidant properties, decreasing the inflammatory burden and oxidative stress [169,177,178].

### 2.3. MetS and Mice Studies

Mouse models can be used to study the pathophysiology by which obesity can lead to the development of IR. Elevations in abdominal circumference and IR have been seen in diet-induced and genetic mouse models of MetS. Many of the mouse models most recognized in Kennedy et al.’s study of obesity arose from spontaneous mutations. Among these are leptin-deficient (Lepob/ob), leptin receptor-deficient (LepRdb/db), and lethal yellow agouti (Ay/a) mice (Table 4) [179]. The abbreviations Lepob/ob and LepRdb/db will be used as they bring in elements of their traditional phenotypic, and their more recently discovered genetic, designations. Although their major phenotype is obesity, these mice also exhibit IR and some degree of dyslipidemia; thus, they can be used as models of MetS. The tub and fat mutations are other spontaneous monogenic forms of obesity seen in mice; however, these mice are less well characterized and are not as commonly used for studies of MetS. Two genetic knockout models of obesity were discussed, the melanocortin 4 receptor (MC4-R) and melanocortin 3 receptor (MC3-R) knockout mice. 

## 3. Management

### 3.1. Mediterranean Diet

Long known to be associated with low risk of cardiovascular disease, the adherence to the Mediterranean diet has also been shown to decrease the risk of MetS [194,195]. It has been shown to have a protective role on every component of the MetS pentad [194]. The antioxidant and anti-inflammatory properties of the different Mediterranean diet components, including olive oil, fish, cereals, vegetables, and fruits, likely constitute the most likely explanation of those findings [196,197,198,199]. A recent metanalysis of 50 prospective studies and randomized control trials showed that the greater the adherence to the Mediterranean diet, the greater the reversion of MetS and its components [200]. 

### 3.2. Nutraceuticals 

Recent research is assessing the role of nutraceuticals in the management of MetS. Several plant extracts, spices, herbs, and essential oil extracts have apparent benefit in the management of patients with MetS. Although some benefits have been documented, these agents are still under investigation and cannot be considered as an alternative for pharmacotherapy. However, the dependence on nutraceuticals—that are graciously available and with minimal complications—in the management of MetS may be a promising field in the development of novel therapies [201]. 

Table 5 illustrates the nutraceuticals that have been studied and have proven some benefits in MetS.

### 3.3. Butyrate

Human gastrointestinal microbiota have been broadly explored because of their role in both maintaining gut health and disease causation. Dietary fiber and resistant starch are not completely hydrolyzed by host enzymes in the small intestine; instead, they are broken down by the large intestinal microbiota. The principal fermentation products resulting from the breakdown of these fibers are the short-chain fatty acids (SCFAs) acetate, propionate, and butyrate. SCFAs are an important fuel for intestinal epithelial cells, and the main energy source is butyrate, followed by propionate and then acetate (butyrate > propionate > acetate). In general, approximately 10% of the daily energy requirement in adults on a westernized diet originates from the colon-derived SCFAs.

Recently, butyrate has been reported to improve lipid profile, glycemia, body weight, composition, and insulin sensitivity in animal models of MetS. In vitro analyses tested the effect of butyrate on adipose tissue, intestinal cells, skeletal muscle, pancreatic islets, hepatocytes, and blood vessels, emphasizing the role of genes and pathways interplay in its beneficial effects. 

A link between obesity and the components of the human microbiota has been described. Nevertheless, these conclusions depend on experimental studies completed using relatively small and distinct groups of volunteers or model animals. Moreover, microbiota components and nutrient intake regulate the total amount and relative quantity of butyrate [216].

A copious and phylogenetically distinct group of bacteria in the colon produce butyrate; the main species are Faecalibacterium prausnitzii, Eubacterium rectale, and Roseburia intestinalis. These bacteria are reduced in diabetic and pre-diabetic subjects [217]. Microbiota taken from lean subjects show increased comparative levels of bacteria that produce butyrate and after transferring them to subjects with MetS, the insulin sensitivity is improved [218]. 

Butyrate mechanisms of action are diverse, and several of these include epigenetic regulation of gene expression by inhibiting histone deacetylase [219]. 

In epigenetics, histone N-terminal tail acetylation is believed to improve gene transcription. This adjustment of histone acetylation and deacetylation by environmental factors, such as dietary compounds, may decrease diseases and maintain good health. Additionally, the inhibition of histone deacetylase (HDAC) and the activation of AMPK by butyrate stimulate PGC-1α activity can occur [220]. By binding and activating the G protein-coupled free fatty acid receptors (FFAR), FFAR2 and FFAR3 in the intestinal enteroendocrine cells, butyrate is believed to play a major role in metabolism and appetite suppression [221].

**Figure 2:**
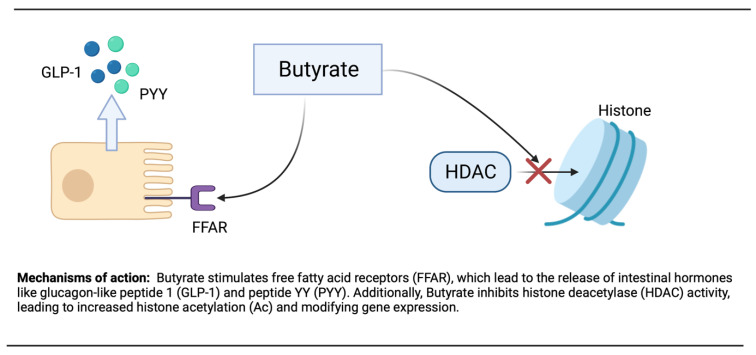
Mechanism of action of butyrate.

Remarkably, butyrate supplementation or high levels of fibers in the diet, as well as increase gut bacteria, have been shown to prevent or attenuate obesity and insulin resistance [222]. By stimulating FFARs in the intestine, butyrate may reduce appetite and weight. The activation of FFARs stimulates the release of glucagon-like peptide 1 (GLP-1) and peptide YY (PYY) (Figure 2). GLP-1 will increase insulin secretion and inhibit glucagon secretion, whereas PYY will reduce appetite and slow gastric emptying [223]. Additionally, butyrate decreases the secretion of ghrelin, the hunger hormone. In animals given high-fat diets (HFD), butyrate supplementation decreased weight gain by reducing increases in adiposity. Matheus et al. [224] showed that butyrate did not modify weight, adiposity, glycemia, or insulinemia in mice on a non-HDF, whereas notably these paramters are ameliorated in mice on an HFD, indicating that butyrate improves the unfavorable effects of an HFD. The beneficial effects seen with butyrate supplementation can also be observed with fermentable fibers and resistant starches [225].

As mentioned previously, the impact of butyrate on intestinal cells, adipose tissue, skeletal muscle, blood vessels, hepatocytes, and pancreatic islets has been studied by accentuating the role of different genes and pathways.

The effect of butyrate on adipose tissue has been described in several studies, revealing that butyrate activates adipogenesis by several mechanisms, mainly by increasing the expression of genes that play major roles in adipocyte differentiation [226]. Butyrate markedly stimulated the expression of SREBP-1c’s mRNA, a major regulator for adipogenesis and fatty acid de novo synthesis. Moreover, the expression of the adipocyte’s differentiation markers, PPARγ, C/EBPα, and C/EBPβ, were significantly upregulated in adipocytes treated with butyrate compared to the control group. Increased adipocytes lead to more lipids being sequestered, thus more adipogenesis reduces circulating fatty acids and hence protects different organs from lipotoxicity and ensuing metabolic disorders such as insulin resistance [227].

As for cholesterol metabolism, several animal studies have revealed a decrease in total serum cholesterol level when given butyrate. This reduction in total serum cholesterol may be due to modifications in dietary cholesterol uptake, cholesterol import, and export from cells or cholesterol biosynthesis. These three processes are proposed in in vitro studies. In a study conducted by Marcil et al. [228], butyrate was found to reduce cholesterol synthesis in Caco-2 enterocytes, and this process is related to a decrease in the activity of 3-hydroxy-3-methyl-glutaryl-coenzyme A (HMG-CoA) reductase (HMGCR), a rate-limiting step in the cholesterol biosynthesis. Additionally, a study of Caco-2 cells described the role of butyrate in reducing the absorption of cholesterol by intestinal cells and decreasing the expression of a major gene in intestinal cholesterol absorption [229]. Moreover, the gene expression of ABCG5 and ABCG8, implicated in moving cholesterol from intestinal cells into the intestinal lumen and therefore away from the circulation, was enhanced. These outcomes were not seen in cells treated with propionate or acetate, signifying this is not a conventional effect of SCFAs. 

In diabetic rats, Khan and Jena [230] observed lower HBA1c and plasma glucose with unchanged plasma insulin when given butyrate, thus showing that butyrate decreases insulin resistance. Moreover, butyrate may improve insulin sensitivity by promoting adipogenesis. This implies that butyrate can act in a similar way to thiazolidinediones, improving adipogenesis through PPARγ stimulation. An increase in expression of the glucose transporter GLUT4 and hence an increase glucose uptake was observed in porcine adipocytes treated with butyrate [226]. Finally, a study made by Chriett et al. indicated that butyrate increased histone acetylation near the IRS1 promoter, thus amplifying its expression [231]. 

Protecting the pancreatic β cell function is also a major role of butyrate. Sodium butyrate given to a rat model of type 1 diabetes decreased the destruction of pancreatic islets caused by streptozotocin (STZ) injection [232]. 

In terms of endothelial function, hypertension and vascular function were improved after the supplementation of butyrate in a human clinical trial [233,234]. Several mechanisms responsible for blood pressure reduction were hypothesized, including vasodilation, increased nitric oxide production, decreased proliferation of vascular smooth muscle cells, and the renin–angiotensin system. 

Whether results from animal studies can apply to humans is open to question because of butyrate’s poor systemic availability and rapid clearance from the circulation. Studies have shown that the amount of butyrate in the gastrointestinal tract that reaches the peripheral circulation is minimal [233,234]. In fact, it is the main reason why many butyric prodrugs and derivatives have been developed. As an example, Pivanex (pivaloyloxymethyl butyrate, Prodrug AN9) has considerably higher cellular uptake than butyrate because of its lipophilicity and is active at lesser concentrations [235]. Nevertheless, several side effects were reported in a phase I trial involving nausea; vomiting; fatigue; visual disturbance; and, captivatingly, hyperglycemia [236].

However, a study on non-human primates has shown increase uptake in specific tissues such as the pancreas and the liver. Thus, oral administration of butyrate may yield positive effects on lipid and glucose metabolism by acting in the liver and pancreas [237].

For the moment, most human clinical trials have focused on the role of butyrate in intestinal disorders administered rectally or orally, and several trials showed improvement in diverticulitis [238], Crohn’s disease [239], ulcerative colitis [240], and travelers’ diarrhea [241]. No major side effects were described, and it was well tolerated. The unique problem was that the orally administered butyrate led to foul-smelling belching [240].

**Figure 3:**
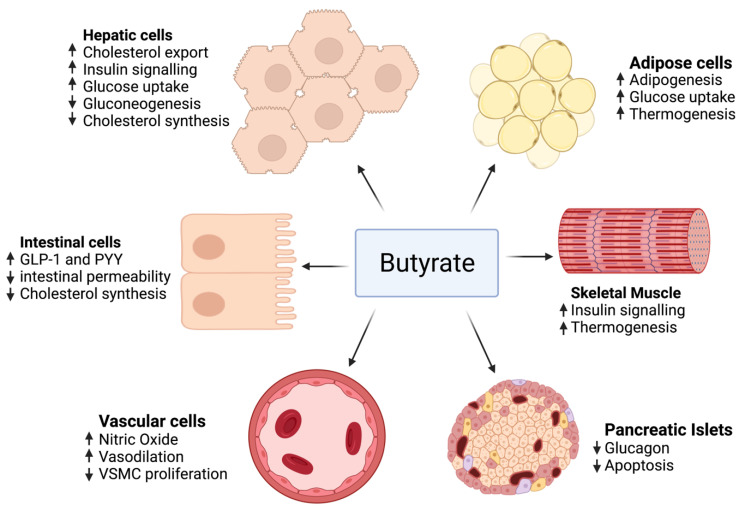
The effect of butyrate on adipose tissue, intestinal cells, skeletal muscle, pancreatic islets, hepatocytes, and blood vessels.

On the other hand, no sufficient human clinical trials studied butyrate benefits in MetS. Only two small trials were conducted. One was a randomized placebo-controlled clinical trial that studied the outcome of 100 mg sodium butyrate given six times daily for 45 days in type 2 diabetics [233], finding that there was no improvement in fasting insulin, fasting blood glucose, cholesterol, or triglyceride levels; however, an increase in GLP-1 and a reduction in diastolic blood pressure were observed. The second trial used a small sample size and was not placebo-controlled. It studied the effect of 4 g of oral sodium butyrate given daily for 4 weeks. The outcome was an improvement of hepatic and peripheral insulin sensitivity in lean males rather than obese males [242]. Figure 3 summarizes the role of butyrate in MetS.

### 3.4. Probiotics

Nowadays, there is evidence that gastrointestinal dysbiosis, which is the modification of the gastrointestinal microbiome, can participate in insulin resistance development [243]. Viewed in this way, many studies have revealed the undesirable effects of low or atypical gastrointestinal bacterial load on dyslipidemia, inflammatory markers, adiposity, and insulin resistance [244]. Additionally, one of the mechanisms was exposure to the bacterial LPS, which is the main cause of metabolic endotoxemia linked to MetS [245]. Moreover, studies have shown that compared to normal-weight people, obese individuals have a reduced ratio of Bacteroidetes/Firmicutes [246].

In terms of therapy, several studies have evaluated the beneficial effects of probiotics in obese and MetS patients (Table 6). Analysis of the data shows that the inclusion of probiotics to the treatment of patients with MetS may improve the current medical treatment but would not affect all of the clinical characteristics of the MetS and related inflammatory biomarkers. Although we cannot deduce that probiotics exert beneficial effects, they may have some positive outcomes that are marginal compared to drug therapy and a healthy lifestyle, and are likely dose- and strain-specific. In addition, the duration of the interventions may have not been long enough to prove the decrease in MetS associated complications. Accordingly, better RCTs were needed in humans with MetS able to fully enlighten if probiotics can be truly used as a coadjuvant therapy for this pathology.

Nine clinical trials are highlighted in the following section. Six of them were performed in both men and women, whereas three of them were performed in postmenopausal women. The dose used and the duration of the intervention varied from 10^8^ cells/mL to 1.5 × 10^11^ colony-forming units (CFU)/g, and from 3 to 12 weeks, respectively. The main results for primary and secondary outcomes, specific strains used, and characteristics of the studies are described in the table below (Table 6) [245,246,247,248,249,250,251,252,253].

Interestingly, one specific strain (Lactobacillus reuteri V3401) has gained attention in the literature and has shown encouraging results in animal studies. Thus, a randomized, placebo-controlled, crossover study was conducted on 53 adults with MetS [255], and the participants were divided among two groups: one group received the probiotic Lactobacillus reuteri V3401 for 12 weeks concurrently with hypocaloric diet and physical activity and the other group received a placebo, also with a healthy lifestyle recommendation. No differences were observed regarding clinical characteristics of MetS in the group treated with Lactobacillus reutri V3401; however, changes in inflammatory biomarkers, anthropometric measurements, and microbiome composition were reported: reduction in IL-6 and VCAM-1 levels, improvement in anthropometric parameters, and increased proportion of Verrucomicrobia phylum [255]. 

Results regarding the utility of probiotics in the treatment of MetS have been contradictory. These conflicting results are probably due to the diverse doses, strains, and study designs [247]. Hence, more studies are needed to confirm the beneficial effects of probiotics and further explore their mechanism of action.

### 3.5. Coconut Oil Effect on Asymmetric Dimethylarginine (ADMA)

Diet has been shown to be one of the most crucial factors that contribute to MetS. Additionally, dietary fats are well known to affect lipoprotein and triglyceride levels [256]. Therefore, many studies have evaluated the effect of dietary fat on MetS. Some of them suggested that reducing dietary fat had a beneficial effect [257]. However, several other studies indicated that the type of fats that an individual consumes is more essential than the quantity of fat for the treatment of MetS [258]. 

Saturated and trans fatty acids are generally considered to have negative effects on cardiovascular risk, and recent studies have advised reducing their consumption to no more than 10% of total calories daily [259]. Conversely, medium-chain fatty acid (MCFAs) and SCFA consumption showed a beneficial effect on MetS [260].

Lately, several studies have evaluated the beneficial effect of coconut oil as an antithrombotic, anti-inflammatory, and hypolipidemic agent [261]. Coconut oil has an elevated quantity of MCFAs—it is made of 8% caprylic acid, 7% capric acid, 8% myristic acid, 2% stearic acid, 6% oleic acid, 8% palmitic acid, 2% linoleic acid, and 49% lauric acid [262]. Although 85% of coconut oil is composed of saturated fatty acid (SFA), the major SFA, lauric acid, has been shown to have positive health outcomes in CVD risk profile and body composition when compared with other SFAs such as palmitic acid, which exists mainly in palm oil, butter, and animal fat [263]. Additionally, when compared with refined coconut oil, virgin coconut oil (VCO) has a more antioxidant effect because of the high proportion of phenolic content. Consequently, by decreasing oxidative stress, it has further benefits in preventing cardiovascular dysfunction [261].

ADMA, which is a product of methylated protein metabolism in the body, plays a major role in endothelial dysfunction (ED) by inhibiting NO synthase [264]. Many consider endothelial dysfunction (ED) as part of the MetS because it is an early stage of atherosclerosis [265]. Moreover, elevated serum levels of ADMA in MetS have been described [265,266]. Consequently, ADMA can be a target to prevent adverse cardiovascular outcomes in MetS. Several studies have shown that consumption of high-fat meals is associated with an increased level of ADMA [266]. Yet, little is known about the effect of the composition of dietary fat on ADMA blood levels.

The latest review emphasized on the need for more studies on the benefits of VCO on dyslipidemia and CVDs risk factors. Thus, in a study conducted by Parinaz Nikooei et al., 30 mL of VCO was given for 4 weeks, replacing the daily used oil. Its effect on MetS components as well as on serum ADMA levels were investigated and showed that consuming 30 mL of VCO for 4 weeks instead of the habitual daily oil decreased serum fasting blood glucose and triglyceride and increased HDL-C, total cholesterol, LDL-C, and ADMA. However, the ratio of LDL-C/HDL-C did not change. Additionally, there were no beneficial effects on waist circumference and blood pressure. Further studies are needed to cautiously recommend VCO, specifically in people at risk for CVDs [267]. 

### 3.6. Curcumin

Curcumin, a polyphenolic compound, is an orange-yellow pigment that is derived from turmeric [268]. Its beneficial effects have been studied widely. It has an anti-oxidative effect, as well as anti-inflammatory properties, and increases nitric oxide production [269]. These effects may help improve arterial stiffness caused by inflammation and increased luminal pressure. A study conducted by Alidadi et al. revealed that consuming 500 mg of curcumin every day for 12 weeks has beneficial effects on arterial stiffness and can control the weight of individuals with MetS [270]. After 12 weeks of curcumin administration, there was a statistically significant decrease in mean body weight in the control compared to the placebo group; however, body composition and other anthropometric parameters revealed no major changes. Curcumin supplementation showed no effect on vascular stiffness and hemodynamic parameters at the baseline of the study. However, after 12 weeks of curcumin supplementation, a significant decrease in PWV was observed, with amelioration of aortic stiffness in MetS patients. Even after adjusting for confounding factors, including gender, age, change in physical activity, and energy intake by regression, the daily consumption of curcumin significantly lowered aortic PWV, relative to placebo [270]. Moreover, in terms of diabetes and dyslipidemia, fasting blood glucose did not improve in the group treated with curcumin, and lipid profiles remained unchanged. However, preclinical studies have shown that curcumin reduces serum cholesterol levels by upregulation of hepatic LDL receptor expression and inhibition of LDL oxidation [271]. Gene expression involved in cholesterol biosynthesis is also affected by curcumin, as well as cholesterol excretion by increasing bile acid secretion [271]. In addition, it is believed that curcumin contributes to the downregulation of major lipogenesis and adipogenesis transcription factors such as PPARγ [272]. Thus, to explain the discrepancy in results, it has been postulated that to affect lipid profiles and fasting plasma glucose in individuals with MetS, a higher dose or higher efficacy form of curcumin should be taken [273].

Preliminary evidence in a preclinical analysis completed by Fleenor et al. proved that dietary curcumin supplementation reduces age-related large elastic artery stiffness by restoration of nitric oxide bioavailability, reduction of oxidative stress, and regulation of collagen I and advanced glycation end products buildup in the arterial wall [274].

### 3.7. Statins

The role of the proinflammatory and prothrombotic state in MetS has been investigated by several studies. Statins have been proven to reduce high-sensitivity C-reactive protein (hs-CRP) blood levels, confirming their anti-inflammatory effect. 

In a study conducted by Gladys P. Velarde et al., women with MetS were administered high-intensity atorvastatin for 3 months, and several markers were reviewed, such as lipogenesis, inflammation, and thrombogenesis [275]. Of note, women in the study were not affected by cardiovascular disease or diabetes and had minor 10 year ASCVD risk. Results showed that atorvastatin led to the reduction of total cholesterol, LDL-C, and Apo-B levels as well as the Apo-A1/Apo-B ratio. However, no effect was shown on Apo-A1, human leptin levels, and lipogenic markers. Compared to placebo, there was no meaningful impact of atorvastatin on myeloperoxidase and plasminogen activator inhibitor-1 levels, and no major effect on fasting blood glucose (FBG) and microalbumin level. Moreover, no conclusion was made regarding platelet aggregation in response to adenosine diphosphate and collagen by treatment. 

Adding up to the expected effect of atorvastatin on lipid profile, a significant reduction in Apo-B levels and Apo-B/Apo-A1 ratio and increase VCAM-1 levels was detected. No significant effect on Apo-A1, human leptin, hs-CRP, ICAM-1 was noticed. However, CRP levels were monitored in a different study and appeared to be decreased after statin therapy, independently of LDL cholesterol [276,277].

Interestingly, in a study conducted by de Oliveira et al. in 2014, simvastatin alone, pioglitazone alone, as well as their combination were shown to decrease the epicardial fat and the inflammatory markers in patients with coronary artery disease and MetS [278].

### 3.8. Anti-Hyperglycemic Agents

In addition to pioglitazone, mentioned above, dipeptidyl peptidase-4 (DPP-4) inhibitors have also been studied in patients with MetS. Multiple studies have suggested that the DPP-4/incretins axis has an influence on the cardiovascular system [279,280]. In addition, DPP-4 expression was shown to positively correlate with the extent of central obesity, visceral adiposity, and inflammation [281,282]. Sitagliptin, one of the DPP-4 inhibitors, has been shown to decrease MetS, obesity-induced adipose tissue inflammation, and fatty liver by regulating the adenosine monophosphate-activated protein kinase and adiponectin levels in obese mice [283]. Moreover, sitagliptin has been shown to have a positive influence on hyperglycemia-induced vascular changes in an endothelium-dependent manner [284]. Finally, sitagliptin has been shown to decrease visceral adiposity as well as the maximal response to oral glucose tolerance test in women with polycystic ovarian syndrome. Glucagon-like peptide 1 (GLP-1) receptor agonists have become popular and more widely used in the past couple of years. Rizzo et al. recently highlighted the importance of utilizing GLP-1 receptor agonist and their effect on various cardiometabolic markers and overall cardiovascular risk [285]. Even though the exact mechanisms remain largely unknown, the favorable effects of liraglutide, a GLP-1 receptor agonist, appear to be driven by direct anti-atherosclerotic action and by decreasing plaque formation and progression [286].

The Diabetes Prevention Program (DPP) addresses in the post hoc analyses of their clinical trial the possibility of modifying MetS prevalence estimates after treatment with either lifestyle (exercise-induced weight loss + diet) or metformin [287]. They reported the incidence of new MetS cases and the resolution of existing MetS cases in treatment groups compared with placebo in participants (*n* = 3234) of the DPP trial after 3.2 years of follow-up. However, since the impaired glucose tolerance (IGT) was a primary inclusion criterion in the trial, 53% of the participants met the criteria of MetS at baseline. Compared to placebo, the incidence of MetS was decreased by 41% in the lifestyle group and by 17% in the metformin group [287]. Among those who fulfilled MetS criteria at baseline, prevalence at 3 years varied significantly by treatment group: 38% of the lifestyle group, 23% of the metformin group, and 18% of the placebo group no longer had the syndrome [287]. Whether these effects apply to non-IGT population remains unknown. Several efforts have been put in pace to answer this question [288]. 

## 4. Conclusions

A cluster of components or risk factors are associated with an increased risk of CVDs and T2DM, and MetS has a prevalence that has been on the rise across all ages. While it is not clear if MetS can be treated in and of itself, ongoing research seems to be unlocking different targets along the disease’s pathway. Concentrating therapeutic efforts on treating the excess adiposity and insulin resistance associated with the MetS may provide the most overall success in attaining these goals. Much has been answered in the past few years, but much remains to be unraveled. The associations of MetS with other comorbidities such as NAFLD, sleep disorders, reproductive tract disorders, and microvascular disease are examples of areas yet to be fully determined.

## Figures and Tables

**Table 1:** Evolution of metabolic syndrome diagnostic definitions throughout the years.

Clinical Measure	Criteria					Diagnosis
	Central Obesity	Blood Glc	High TG	Low HDL	High BP	
AHA/ NHLBI (2009) [4]	WC >40” (men) or >35” (women)	IFG or on high blood Glc txt or T2DM dx	≥150 mg/dL or on TG txt	<40 mg/dL (men) or <50 mg/dL (women) or on HDL txt	≥130 mmHg systolic and/or ≥85 mmHg diastolic or on HTN txt	≥3 criteria
IDF (2005) [5,6]	WC >37” (men) or >32” (women) or BMI >30 kg/m^2^					≥3 criteria one of which should be central obesity
ATPIII (2001) [7]	WC >40” (men) or >35” (women)		≥150 mg/dL	<40 mg/dL (men) or <50 mg/dL (women)	≥130 mmHg systolic and ≥85 mmHg diastolic or on HTN txt	≥3 criteria
EGIR (1999) [8]	WC >37” (men) or >32” (women)	IFG or IGT		<39 mg/dL (men and women)	≥140 mmHg systolic and ≥90 mmHg diastolic or on HTN txt	≥3 criteria one of which should be IR *
WHO (1998) [1]	Waist/hip ratio > 0.9 (men) or > 0.85 (women) or BMI > 30 kg/m^2^	IFG orx IIGT or T2DM dx		<35 mg/dL (men) or <39 mg/dL (women)	≥140 mmHg systolic and ≥90 mmHg diastolic	≥3 criteria one of which should be IR **

Note that IFG is defined as ≥110 mg/dL in 2001 but this was momdified in 2004 to be ≥100 mg/dL, IGT is defined as 2 h glucose >140 mg/dL. * EGIR IR is defined as plasma insulin levels >75th percentile. ** WHO IR is defined as presence of IR or IFG or IGT. Abbreviations: AHA: American Heart Association, ATPIII: National Cholesterol Education Program Adult Treatment Panel III; dx: diagnosis; EGIR: European group for study of insulin resistance; Glc: glucose; HDL: high density lipoprotein; HTN: hypertension; IR: insulin resistance; IDF: International Diabetes Federation; IGT: impaired glucose tolerance; IFG: impaired fasting glucose; NHLBI: National Heart, Lung, and Blood Institute; TG: triglyceride; txt: treatment; WC: waist circumference; WHO: World Health Organization.

**Table 2:** HOMA-IR and QUICKI cut off value.

Index	AUC (95% CI)	Optimal Cut off Point	Sensitivity	Specificity
HOMA-IR				
Men MetS	0.7000 (0.68034–0.71972)	2.00	64.4	66.8
Women MetS	0.6779 (0.65530–0.70043)	2.50	57.6	67.9
QUICKI				
Men MetS	0.7016 (0.68198–0.72129)	0.343	63.7	67.8
Women MetS	0.6803 (0.65779–0.70281)	0.331	55.7	70.7

**Table 3:** Important inflammatory biomarkers and adipokine dysregulation in MetS. ↑ signifies increase. ↓ signifies decrease.

Increased Expression (↑)	Decreased Expression (↓)
↑ Leptin, PAI-1, chemerin ↑ IL-1, IL-6, IL-8, MCP-1, TNF-α ↑ High sensitivity CRP, fibrinogen ↑ Monocytic TLR2 and TLR4	↓ Adiponectin, omentin ↓ IL-10

**Table 4:** Latest studies revolving around MetS in mice. ↑ signifies increase. ↓ signifies decrease.

Model	Obesity	Hyperlipidemia	IR	Hypertension	Caveats	Reference
OBESITY MODELS						
Lepob/ob	From weaning	F HDL (LDL/HDL1)	Yes	↑↓ blood pressure	No leptin signalling; strain differences; anomalies with reproduction, thyroid axis, and HPA axis	[180]
LepRdb/db	From weaning	F HDL (LDL/HDL1)	Yes	↑↓ blood pressure	No leptin signalling; strain differences; anomalies with reproduction, thyroid axis, and HPA axis	[181]
Ay/a	Postponed onset	Slight F HDL	Postponed	yes	Tumor formation	[182]
MC4-R–/–	Postponed onset; exacerbated following high-fat feeding; haploinsufficiency of MC4-R also seen in obese humans	ND	Yes	↓ blood pressure	-	[183]
MC3-R–/–	Postponed onset	ND	protected	ND	↑ adiposity without an increase in body weight	[184]
HYPERLIPIDEMIA MODELS						
LDLR–/–	HFD induced	↑ LDL	HFD induced	-	-	[185]
apoE–/–	Generally resistant	↑ VLDL and LDL, ↓ HDL	Generally resistant	-	-	[186]
OBESITY WITH HYPERLIPIDEMIA MODELS						
Lepob/ob;LDLR–/– and LepRdb/db;LDLR–/–	From weaning	↑↑ VLDL and LDL	Yes	ND	Extreme hyperlipidemia; no leptin signaling	[187]
Lepob/ob;apoE–/– and LepRdb/db;apoE–/–	From weaning	↑↑ VLDL and LDL, ↓↓ HDL	Yes	ND	Extreme hyperlipidemia; no leptin signaling	[187]
Ay/a;LDLR–/– Western diet feeding	Postponed onset	↑ VLDL and LDL	Yes	ND	Extreme hyperlipidemia	[188]
LDLR 3KO	From weaning	↑ VLDL and LDL	Yes	Yes	Extreme hyperlipidemia; no leptin signaling	[189]
ApoE 3KO	From weaning	↑ VLDL and LDL	Yes	Yes	Extreme hyperlipidemia; no leptin signaling	[190]
ApoE–/– 60% HFD	Over time on HFD	↑ VLDL	Yes	Yes	-	[191]
OBESITY WITH HYPERTENSION MODELS						
NZBWF1	Age onset	ND	Yes	Yes	-	[192]
KKAy/a	yes	-	Yes	Yes	-	[193]

ND = not determined; HFD = high-fat diet.

**Table 5:** Role of nutraceuticals in MetS. ↑ signifies increase. ↓ signifies decrease.

Source	Action
Turmeric (*Curcuma longa*) Active ingredient: diferuloylmethane in curcumin	Suppress NF-kB activation-> ↓ expression of pro-inflammatory cytokines-↓ TNF-α expression, ↓ expression of plasminogen activator inhibitor type-1-> ↓ inflammation [202]—antioxidant effect [201]—curcumin hinders Wnt/β-catenin pathway associated with obesity [203]
Garlic (*Allium sativum*) Active ingredient: allicin	Anti-inflammatory effect from the organosulfur compounds in its derivatives. Antioxidant action due to thiol groups—Antithrombotic effect [201]—increases insulin sensitivity [204]
Cinnamon (*Cinnamomum verum*) Active ingredient: polyphenols	Antithrombotic–antioxidant–anti-inflammatory effects—increases insulin sensitivity—regulates blood glucose and blood pressure [201]
*Rhizoma coptidis* Active ingredient: berberine	Improves body weight, triglyceride levels—increases insulin sensitivity—downregulation of genes involved in lipogenesis [205]—reduction in blood pressure [206]
Neem (*Azadirachta indica*) Active ingredient: neem oil	Increases glucose tolerance via reduction of intestinal and pancreatic glucosidase activity → improves post-prandial hyperglycemia [207]—regenerates pancreatic beta cells → ↑ insulin secretion [201]
Bergamot orange (*Citrus bergamia*) Active ingredient: bergamot essential oil	Anticancer–anti-inflammatory–antimicrobial–antioxidant–antianxiety properties—↓ ROS formation—↓ lectin-like LDL receptor-1 expression [208]
Grapes (*Vitus vinifera*) Active ingredient: resveratrol, (3,5,4′-trihydroxystilbene)	↓ Adipogenesis—↑ lipolysis—inhibits cyclooxygenase → antioxidant action [209]—enhances insulin sensitivity, glucose tolerance, overall weight, and BMI—[210]
Onions (*Allium cepa*) Active ingredient: quercetin	Anti-inflammatory—antioxidant—↓ blood pressure—↓ cholesterol levels—↓ insulin resistance [211]
Fish oils (omega fatty acids) Active ingredient: polyunsaturated fatty acids	↓ Lipogenesis—↑ fatty acid oxidation in liver and adipose tissue—regulates peroxisome proliferator—activates receptor gamma [212]
Broccoli (*Brassica oleracea*) Active ingredient: sulforaphrane	Anti-inflammatory properties—activates nuclear factor erythroid 2-related factor 2, an antioxidant transcription factor → antioxidant properties—role against hypertension, hyperlipidemia, and diabetes [213]
Ginger Active ingredient: gingerols, shogaols, parasols	Anti-inflammatory—↓ cyclooxygenase-2—↓ 5-lipoxygenase—↓ systolic blood pressure [201]
Cumin (*Cuminum cyaminum*) Active ingredient: cuminaldehyde	↓ Lipid levels—↓ glycemia [201]
*Cynara cardunculus* (L.) subsp. scolymus Hayek-based Altilix^®^	Modulates the expression of PPAR-γ and inhibits fatty acid synthase activity—↓ in body weight, waist circumference, HbA1c, plasma lipids, hepatic transaminases, flow-mediated dilation, carotid intima-media thickness [214].
Monascus purpureus, red yeast rice	Reversible inhibition of 3-hydroxy-3-methyl-glutaryl-CoA reductase [215]

**Table 6:** Association between metabolic syndrome and probiotics.

Reference	Sample Size (n)	Age Range Probiotic Strain Period of Intervention (Weeks) Probiotic Dose	Primary Outcomes	Secondary Outcomes
[247]	28	Control group: 54.5 ± 8.9 Probiotic group: 51.5 ± 11.4 Lactobacillus casei Shirota 12 milk (65 mL bottles × 3/day) 10^8^ cells/mL	No changes were found in BMI, BP, waist circumference, triacylglycerols, TC, and fasting glucose levels.	High-sensitive CRP (1.86 mg/L in the probiotic group vs. −1.60 mg/L in the placebo group, *p* = 0.016) and LBP levels (5827 ng/mL in the probiotic group vs. −1510 ng/mL in the placebo group, *p* = 0.023) increased within the probiotic group
[248]	40	Control group: 51.7 ± 12.1 Probiotic group: 52 ± 10.9 Lactobacillus plantarum TENSIA 3 cheese (50 g/day) 1.5 × 10^11^ CFU/g	BMI was significantly reduced in the probiotic group (BMI variation in probiotic group −2 vs. −1.6 kg/m^2^ in the placebo group, *p* = 0.031).	A positive association was detected between TENSIA colonization and the extent of change of morning diastolic BP (r = 0.617, *p* = 0.0248)
[249]	28	Control group: 55 ± 9 Probiotic group: 51 ± 11 Lactobacillus casei Shirota 12 milk (65 mL bottles × 3/day) 10^8^ cells/mL	No changes were found in BMI, fasting plasma glucose levels, and HOMA-IR index.	Probiotic supplementation resulted in a significant reduction in sVCAM-1 level (−195 ng/mL in the probiotic group vs. 30 ng/mL in the placebo group, *p* = 0.008) and a significant increase in high-sensitive CRP level (1.86 mg/L in the probiotic group vs. −1.60 mg/L in the placebo group, *p* = 0.002)
[247]	24	Control group: 63 ± 7.6 Probiotic group: 62 ± 4.35 Lactobacillus plantarum 12 milk (80 mL bottles × 1/day) 10^7^ CFU/g	Glucose levels showed a significant reduction in the FM group compared with the NFM group (glucose variation in FM −10.5 vs. −3 mg/dL in NFM group, *p* = 0.037).	Homocysteine levels showed a significant reduction in the FM group compared with the NFM group *p* = 0.019).
[250]	28	Control group: 55 ± 9 Probiotic group: 51 ± 11 Lactobacillus casei Shirota 12 milk (65 mL bottles × 3/day) 10^8^ cells/mL	No changes were found in BMI, BP, waist circumference, triacylglycerols, and TC blood levels.	LcS administration was associated with subtle microbiota changes at a genus level (enrichment of Parabacteroidetes)
[251]	51	No data Bifidobacterium lactis HN019 6 milk (80 mL bottle × 1/day) 3.4 × 10^8^ CFU/mL	Significant differences in BMI variation (probiotic group −1.3 vs. −0.3 kg/m^2^ in control group, *p* = 0.017); TC variation (probiotic group −15 vs. 6 mg/dL in control group, *p* = 0.09) and LDLc variation (probiotic group −17.5 vs. −2 mg/dL in control group, *p* = 0.08).	Significant decrease in TNFα and IL−6 (*p* < 0.05) in the probiotic group
[252]	81	Control group: 58.72 ± 7.25 Low-dose group: 56.38 ± 6.55 High-dose group: 55.16 ± 6.87 Bifidobacterium bifidum W23, Bifidobacterium lactis W51, Bifidobacterium lactis W52, Lactobacillus acidophilus W37, Lactobacillus brevis W63, Lactobacillus casei W56, Lactobacillus salivarius W24, Lactococcus lactis W19, and Lactococcus lactis W58 12 lyophilisate powder low dose (2.5 × 10^9^ CFU/day) or high dose (1 × 10^10^ CFU/day)	Significant differences were found in glucose variation (HD vs. placebo −0.61 mg/dL, *p*= 0.0272; HD vs. LD −0.72 mg/dL, *p* = 0.0043), insulin (HD vs. placebo −0.83 UI/L, *p* = 0.0002; HD vs. LD −0.40 UI/L, *p* = 0.0155), and HOMA-IR (HD vs. placebo −0.90, *p* = 0.0005; HD vs. LD −0.54 mg/dL, *p* = 0.0127).	Significant differences were found in uric acid (HD vs. placebo −0.73 mmol/L, *p* = 0.0109; HD vs. LD −0.92 mmol/L, *p* = 0.0016) and LPS levels (HD vs. placebo −0.99 ng/mL, *p* = 0.001)
[253]	81	Control group: 58.72 ± 7.25 Low-dose group: 56.38 ± 6.55 High-dose group: 55.16 ± 6.87 Bifidobacterium bifidum W23, Bifidobacterium lactis W51, Bifidobacterium lactis W52, Lactobacillus acidophilus W37, Lactobacillus brevis W63, Lactobacillus casei W56, Lactobacillus salivarius W24, Lactococcus lactis W19, and Lactococcus lactis W58 12 lyophilisate powder low dose (2.5 × 10^9^ CFU/day) or high dose (1 × 10^10^ CFU/day)	No changes were found in BMI and BP.	Significant differences were found in the pulse wave analysis systolic pressure (HD vs. placebo −1 mmHg, *p* = 0.0054; HD vs. LD −0.91 mmHg, *p* = 0.0057), the pulse wave analysis augmentation index (HD vs. placebo −0.55, *p* = 0.0079), the pulse wave velocity (HD vs. placebo −0.82 m/s, *p* = 0.0045; HD vs. LD −0.55 m/s, *p* = 0.0189), VEGF (HD vs. placebo −1.09 pg/mL, *p* = 0.0001; HD vs. LD −1.10 pg/mL, *p* = 0.0007), TNFα (HD vs. placebo −1.03 pg/mL, *p* = 0.0009; HD vs. LD −0.68 pg/mL, *p* = 0.0471), and thrombomodulin levels (HD vs. placebo −0.78 ng/mL, *p*= 0.0194)
[254]	44	Control group: 44.55 ± 5.70 Probiotic group: 44.05 ± 6.60 Lactobacillus acidophilus La5, Bifidobacterium lactis Bb12 8 yogurt containing 6.45 × 10^6^ CFU/g of L. acidophilus and 4.94 × 10^6^ CFU/g of B. lactis Bb12	Consumption of probiotic yogurt resulted in a significant reduction in the level of blood glucose (mean difference: −3.80, *p* = 0.01).	Consumption of probiotic yogurt resulted in a significant reduction in the level of VCAM-1 (mean difference −463.39, *p* = 0.001)

BMI: body mass index, BP: blood pressure, CFU: colony-forming units, CI: confidence interval, CRP: C-reactive protein, FM: fermented milk, HD: high dose, HOMA-IR: homeostasis model assessment–insulin resistance, IL-6: interleukin-6, LBP: lipopolysaccharide-binding protein, LcS: Lactobacillus casei Shirota, LD: low dose, LDLc: low-density lipoprotein cholesterol, LPS: lipopolysaccharide, NFM: non-fermented milk, TC: total cholesterol, VCAM-1: vascular cell adhesion molecule 1, VEGF: vascular endothelial growth factor.

## Abbreviations

ACLY	ATP-citrate lyase
ADMA	asymmetric dimethylarginine
AHA	American Heart Association
AHSG	α2-Heremans–Schmid glycoprotein
ALT	alanine aminotransferase
AMPK	AMP-activated protein kinase
Ang II	angiotensin II
Apo-A1	apolipoprotein A1
Apo-B	apolipoprotein B
ASCVD	atherosclerotic cardiovascular disease
AST	aspartate aminotransferase
AUC	area under the curve
BMI	body mass index
CDC	Center of Disease Control and Prevention
CETP	cholesterol ester transfer protein
c-miRNAs	circulatory microRNAs
CRP	C-reactive protein
DAG	diacylglycerol
DAMPs	damage-associated molecular patterns
ED	endothelial dysfunction
ERRs	estrogen-related receptors
ETC	electron transport chain
FA	fatty acids
FADH2	reduced flavin adenine dinucleotide
FFAs	free fatty acids
FFAR	free fatty acid receptors
FTO	fat mass and obesity-associated
GDF15	growth differentiation factor 15
GPCRs	G protein-coupled receptors
GLUT4	glucose transporter
GLP-1	glucagon-like peptide 1
HDAC	histone deacetylase
HDL	high-density lipoprotein
HFD	high-fat diets
HMG-CoA	3-hydroxy-3-methyl-glutaryl-coenzyme A
HMGCR	HMG-CoA reductase
HOMA-IR	homeostatic model assessment-insulin resistance
hs-CRP	high-sensitivity C-reactive protein
ICAM-1	intercellular adhesion molecule 1
IDEFICS	Identification and Prevention of Dietary- and Lifestyle-Induced Health Effects in Children and Infants
IGT	impaired glucose tolerance
IL-6	interleukin 6
IP3	inositol 1,4,5-triphosphate
IRS-1	insulin receptor substrate-1
KO	knockout
LDL-C	low-density lipoprotein
LAP	latency-associated protein
LIKRO	liver-specific insulin receptor knockout
LOX-1	lipoprotein receptor-1
LPS	lipopolysaccharides
MCP-1	monocyte chemoattractant protein-1
MD	Mediterranean diet
MetS	metabolic syndrome
MCFAs	medium-chain fatty acids
miRNAs	microRNAs
NHANES	National Health and Nutrition Examination Survey
NADH	nicotinamide adenine dinucleotide
NADPH oxidase	nicotinamide adenine dinucleotide phosphate oxidase
NAFLD	non-alcoholic fatty liver disease
NF-kB	nuclear factor kappa-light-chain enhancer of activated B cells
NO	nitric oxide
NREP	neuronal regeneration–related protein
NRF-1 and NRF-2	nuclear respiratory factors 1 and 2
PAI-1	plasminogen activator inhibitor 1
PAMPs	pathogen-associated molecular patterns
PGC-1α	peroxisome proliferator-activated receptor-γ coactivator 1α
PI3K	phosphoinositide 3-kinase
PPARα and PPARδ	peroxisome proliferator-activated receptor α and δ
PPARγ	peroxisome proliferator-activated receptor-γ
PPY	peptide YY
PWV	pulse wave velocity
QUICKI	quantitative insulin sensitivity check index
RAS	renin–angiotensinogen system
ROS	reactive oxygen species
SCFAs	short-chain fatty acids
Sd-LDL	small dense LDL
SFA	saturated fatty acid
SNPs	single nucleotide polymorphisms
SREBP-1	sterol regulatory element-binding protein 1
STZ	streptozotocin
T2DM	type II diabetes mellitus
TFAM	mitochondrial transcription factor A
TFB2M	mitochondrial transcription factor B2
TGs	triglycerides
Th1	T helper type 1
TNF α	tumor necrosis factor α
TLRs	Toll-like receptors
VCAM-1	vascular cell adhesion molecule 1
VLDLs	very low-density lipoproteins
VSMCs	vascular smooth muscle cells
VCO	virgin coconut oil
WBCs	white blood cells
